# Impact of the gastrointestinal microbiome and fermentation metabolites on broiler performance

**DOI:** 10.1016/j.psj.2022.101786

**Published:** 2022-02-18

**Authors:** Dana K. Dittoe, Elena G. Olson, Steven C. Ricke

**Affiliations:** Meat Science and Animal Biologics Discovery Program, Department of Animal and Dairy Sciences, University of Wisconsin, Madison, WI 53706, USA

**Keywords:** broilers, gastrointestinal tract, microbiome, fermentation, metabolites

## Abstract

Optimal broiler performance is dependent on several factors such as bird genetics, environment management, and nutrition. The gastrointestinal tract microbial ecology and metabolic activities have long been considered factors contributing to broiler performance responses. However, until recently, it was difficult to define the impact of the gastrointestinal microorganisms on the broiler host. With advances in microbiome sequencing technology, there has been a rapid increase in data generated using both experimental and commercial broiler operations. As the gastrointestinal microbiome data becomes more in-depth, opportunities to link microbiota composition to broiler performance metrics such as broiler growth rate and feed conversion efficiency have emerged. In parallel, with the increased understanding of the microbiota, there has been a shift to modulate the microbiome in order to alter metabolic patterns such as fermentation products. In this review, fermentation pathways and metabolites and the relationship with the microbiome will be discussed. Additionally, this review will connect these patterns and interpretations with current broiler performance data and the potential future directions these relationships could take the broiler industry.

## INTRODUCTION

Commercial poultry production practices have evolved considerably over the past century. This has resulted from improvements in poultry genetics, nutrition, management, and health maintenance, among other factors (Dittoe et al., 2020). Broiler performance and management have benefited tremendously from the targeted nutritional management research. This research has allowed for the improved understanding of the physiology of birds under commercial environmental conditions. Likewise, diet formulation and nutritional management have reached new levels of sophistication and precision as amino acid requirements have become more accurate and feed enzyme blends have become available (Dittoe et al., 2020). Ultimately, as research has advanced, a more systems-based approach has been realized and incorporated into poultry management strategies to optimize bird performance. As this realization has developed, the need to understand more aspects of the bird beyond the traditional nutrition and physiology disciplines of research has emerged. For example, it has become apparent that the gastrointestinal associated lymphoid tissues (**GALT**) have an expansive role altering bird health and performance beyond the base protection it provides from pathogens as knowledge of the immune and GALT activities in the bird's gastrointestinal tract (**GIT**) have become better known (Adedokun and Olojede, 2019). One of the more significant research transformations regarding the avian GIT system has been the development of a more in-depth understanding of the GIT microbial ecology (Yeoman et al., 2012; Oakley et al., 2014; Stanley et al., 2014; Clavijo and Flórez, 2018; Feye et al., 2020a). This understanding has been in part due to the increasing affordability of genomic sequencing, leading to a virtual explosion in applications for all aspects of food and animal production (Ricke et al., 2017; Feye et al., 2020a,b).

Opportunities to address current issues in poultry management using new applications for emerging concerns have become possible, including the involvement of GIT ecology with establishment of pathogens in the GIT and their interaction(s) with the indigenous nonpathogen microbial population (Ricke, 2017, 2021). Feed additives such as prebiotics, probiotics, organic acids, and botanicals to limit and prevent pathogen colonization (Clavijo and Flórez, 2018; Dittoe et al., 2018; Ricke, 2018, 2021) is a further refinement of this interaction. While these studies are relatively straightforward, other areas of interest remain challenging, such as connecting the GIT microbial composition and microbial shifts throughout the bird's life to performance parameters. Likewise, other factors such as the breed of birds and environmental conditions associated with housing can contribute to this complexity, making it difficult to sort out the most likely drivers (Stanley et al., 2013, 2014; Borey et al., 2020). Recent advances in computational capabilities and additional bioinformatic tools have significantly increased the interpretative power of sequence data and identification of the taxonomic and diversity profile characteristics attributable to extrinsic factors. However, a next key step is to connect GIT microbial functionality with microbiome characterization. A better understanding of overall fermentation and metabolism of GIT microbial communities as well as the contributions of individual members is needed to develop a more complete understanding of the interaction between the GIT microbial population and broiler host. The current review discusses general concepts on 16S rDNA-based microbiome sequencing, fermentation and metabolism, and the potential relationships between the GIT microbiome and broiler performance responses.

## THE POULTRY GIT MICROBIOME – GENERAL CONCEPTS

**Figure 1:**
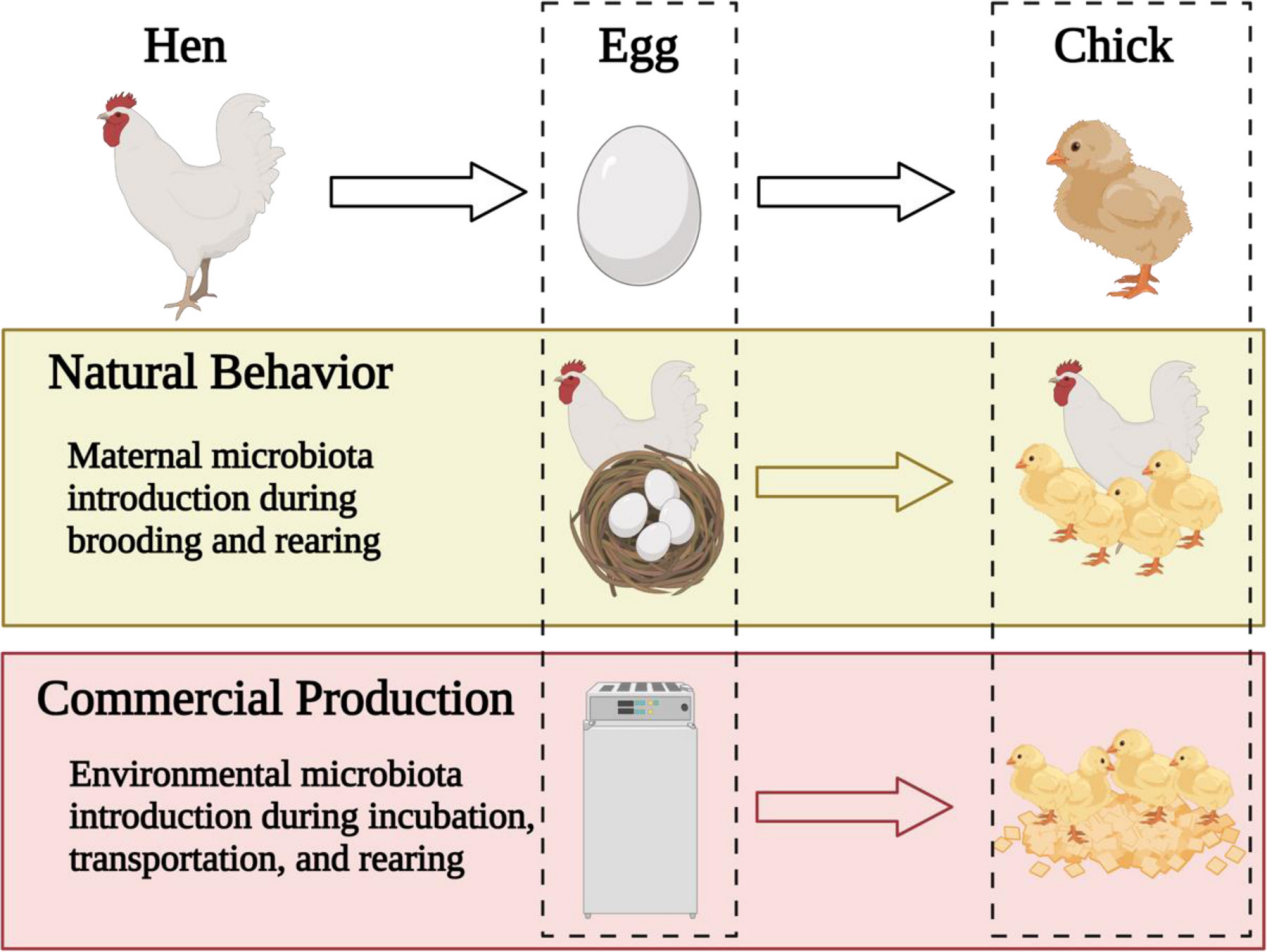
Lack of maternal microbiota introduction in commercial rearing systems has led to the developing GIT microbiota to be highly influenced by the microbiota in the surrounding environment of commercial poultry production such as the hatchery, transportation, and rearing facilities. Figure created with Biorender.com.

The poultry GIT is a dynamic ecosystem with a complex and diverse microbial composition that can be highly variable (Rehman et al., 2007; Stanley et al., 2013, 2014; Feye et al., 2020a). The microorganisms that inhabit the GIT are eventually excreted in poultry feces and can become established in the surrounding environment such as poultry litter and bedding. Compared to the natural or wild rearing conditions in which part of the maternal microbiota is transmitted from the hen to the chick, chickens hatched and reared under large scale commercial operation systems are likely only colonized by the microbiota present in the immediate surrounding environments such as the hatchery, transport, and grow-out facility (house, litter, water, and feed) (Figure 1). Stanley et al. (2013) has concluded that the relatively clean environments in conventional poultry housing hav led to highly variable colonization of bird GIT. Presumably animal and environmental microorganisms potentially interact with each other continuously. The interaction can positively or negatively impact their performance, food safety, and environmental hygiene. Regardless of the presence or absence of pathogens, understanding microbial interactions and monitoring microbial ecosystems is fundamental (Apajalahti et al., 2004; Pan and Yu, 2014; Clavijo and Flórez, 2018).

Interest in understanding and promoting a beneficial intestinal microbiota for maintaining poultry health, limiting foodborne pathogens, and potentially improving poultry performance has increased in the past decades (Apajalahti et al., 2004; Gabriel et al., 2006; Wei et al., 2013; Oakley et al., 2014; Stanley et al 2014; Clavijo and Flórez, 2018; Feye et al., 2020a). This is partly due to improvements in molecular approaches for conducting research on the GIT microbiota. Different molecular techniques, such as G + C profiling, quantitative polymerase chain reaction (**PCR**) single and multiplex assays, and microbial taxa identification through 16S rDNA sequencing, have been used to characterize the broiler intestinal microbiota (Lan et al., 2002; Zhu et al., 2002, 2003; Hume et al., 2003; Amit-Romach et al., 2004; Torok et al., 2008; Yeoman et al., 2012; Yeoman et al., 2012; Wei et al., 2013). Introduction of more advanced Next Generation Sequencing (**NGS**) technologies has led to the identification of individual microbial species, as well as determination of the microbiota's genetic potential, and metabolite-associated activities (Oakley et al., 2014; Stanley et al., 2014; Xiao et al. 2017; Feye et al., 2020a). More specifically, analyses such as targeted amplicon sequencing (DNA and cDNA), metagenomics, and metaproteomics have been used to characterize potential bacterial functionality in targeted environments (Tang et al., 2014; Tilocca et al., 2016). Due to the availability and relatively low cost of more advanced sequencing technology, the in-depth characterizations of GIT microbial responses in real world poultry production settings have now become a reality for both live bird operations and during poultry processing (Stanley et al., 2014; Shaufi et al., 2015; Borda-Molina et al., 2018; Feye et al., 2020a,b).

Studies have also been conducted to characterize chicken cecal functions (Sergeant et al., 2014; Shaufi et al., 2015; Tilocca et al., 2016; Yan et al., 2017; Kumar et al., 2020). Based on conventional poultry production studies, it has become apparent that the GIT microbiota of commercially raised poultry depends on numerous factors such as animal age, environment, and feed composition (Hume et al., 2003; Lu et al., 2003; Gabriel et al., 2006; Feye et al., 2020a). For most poultry, the microbial richness and diversity increases in all GIT compartments as the bird matures (Yeoman et al., 2012; Stanley et al., 2014; Rychlik, 2020). In addition to the cecum, each GIT organ develops its own specific and unique bacterial community over time (Rehman et al., 2007; Wei et al., 2013; Stanley et al., 2014). As such, the GIT microorganisms of a commercial broiler are typically well established by 2 wks of age (Hume et al., 2003; Lu et al., 2003; Rehman et al., 2007; Stanley et al., 2014). As the poultry GIT microbial populations become metabolically more anaerobic, detectable fermentation products become prominent in the sections of the GIT with different profiles occurring as a function of GIT microbial composition and section of the broiler GIT.

## MICROBIAL FERMENTATION PRODUCTS OF THE BROILER GIT MICROBIOTA

The relationship between microbial taxonomic composition and functionality in the poultry GIT has been one of the primary focal research points for developing a better understanding of the poultry GIT microbial ecology. In general terms, the GIT microbial population essentially hydrolyzes dietary components resulting in the formation of either terminal end products or metabolites that other GIT microorganisms can use as substrates. The end product profile may vary widely based upon microbial metabolic pathways, with facultative versus strict anaerobic energy metabolism being quite variable. As the bird matures, the more obligate anaerobic microorganisms begin to emerge and can become established in the cecum which has the most densely populated microbial community of the poultry GIT (Rehman et al., 2007). This microbial population shift can generally influence both the profile and concentrations of detectable end products generated from their fermentative activities. The presence of these end products represents an important role for the poultry GIT microorganisms. For example, end products such as short chain fatty acids (**SCFA**) can be inhibitory to invading foodborne pathogens such as Salmonella and contributes to the bird's energy metabolism (Annison et al., 1968; Ricke, 2003; McWhorter et al., 2009; Sergeant et al., 2014; Dittoe et al., 2018).

Microbial hydrolysis and fermentation of ingested diets produce numerous compounds in the GIT including lactic acid and SCFA (Rehman et al. 2007; Rychlik, 2020). Lactic acid is present in much lower quantities in the small intestine and cecum (Oakley et al., 2014; Shaufi et al., 2015; González-Ortiz et al., 2020). There are several potential causes of this decrease in lactate. Indeed, declines in actual numbers of lactate-producing GIT bacteria occur as the GIT microbial ecology changes with bird maturity (van der Wielen et al., 2000). However, some lactic bacteria are heterofermentative and can switch pathways utilized depending upon substrate availability and other environmental conditions (Russell and Cook, 1995). There may be other reasons as well. For example, in other GIT ecosystems such as the rumen, some GIT microorganisms (e.g., *Selenomonas ruminantium* and *Megaspheara eldsdinii*) can use external lactate as a carbon and energy substrate (Ricke et al., 1996; Chen et al., 2019). It is unknown whether lactate utilizing organisms in the poultry GIT exist and/or can function in this manner. However, poultry *Megaspheara* isolates have been shown to possess over 90% similarity to human *M. elsdenii* (Rychlik, 2020). Likewise, members of the order Selenomonadales which includes the genus *Selenomonas* have been isolated from young broiler chicks (Campbell et al., 2015; Walugembe et al., 2015). Other avenues for lactate utilization may also exist, as Segura-Wang et al. (2021) identified lactate conversion genes for butyrate production in broiler GIT contents.

A wide range of microorganisms located throughout the poultry GIT from the crop to the ceca produce SCFA, including unbranched (acetate, propionate, and butyrate) as well as branched SCFA (valerate, isovalerate, and isobutyrate) with acetate generally being the predominant SCFA (Rehman et al., 2007). The appearance of detectable levels of SCFA occur relatively early in a broiler chick's life and increase as the GIT microbial population proliferates, and more anaerobic bacteria become established (Ricke et al., 1982; van der Wielen et al., 2000; Rehman et al., 2007; Stanley et al. 2014; Walugembe et al., 2015). Overall, the presence of these acids in the GIT is considered unfavorable to coliforms and most other transient pathogens, and their production has been one of the mechanisms attributed to probiotics and prebiotics for eliciting inhibitory activities against the microorganisms (Fuller, 1984; van der Wielen et al., 2000; Ricke 2003; Dittoe et al., 2018). In the crop and the cecum, acetic acid is the dominant SCFA (Fuller, 1984; van der Wielen et al., 2000; Rehman et al., 2007). The evidence for the cecum is supported by metagenomic analyses where over 30 acetate kinase and phosphotransferase sequences were identified in the ceca of 42-day-old broilers (Sergeant et al., 2014).

In addition to the acetic acid levels that have been detected, butyrate and propionate are also prominent SCFA occurring in the poultry GIT, albeit usually in lower concentrations than acetate (Fuller, 1984; Rehman et al., 2007). Butyrate concentrations appear to vary considerably, but the highest concentrations typically occur in the ceca in both young broilers and older birds and this SCFA has been promoted as a feed additive (van der Wielen et al., 2000; Bedford and Gong, 2018). The genes for butyrate from acetyl-CoA and butyrate kinase have been identified in broiler GIT microbial populations (Sergeant et al. 2014; Segura-Wang et al., 2021). Propionate formation by the succinate pathway also occurs primarily in the ceca and the genes for generating propionate via methylmalonyl-CoA mutase, methylmalonyl-CoA decarboxylase, and methylmalonyl-CoA epimerase have been identified in the poultry GIT in the Bacteroidota and Verrucomicrobiota phyla (Sergeant et al., 2014; Segura-Wang et al., 2021). The succinate-propionate pathway has been linked with lactate utilization by rumen selenomonads (Ricke et al., 1996), thus raising the possibility of whether this occurs in the chicken ceca. However, depending upon the biosynthetic capabilities of the GIT microorganism, formation of propionate can be influenced by presence of other metabolites, such as cofactors required for the pathway. For example, the prominent rumen and swine microorganism *Prevotella ruminicola* and human isolates of *Bacteroides* in the absence of external vitamin B_12_, produce succinate rather than propionate due to their inability to synthesize B_12_ (Chen et al., 1981; Strobel, 1992). Whether this occurs with poultry GIT microorganisms is not known, although presumably B vitamin supplementations in broiler diets would probably mask this bacterial biosynthetic deficiency. Under certain conditions, such as administration of propionate generating competitive exclusion cultures, the concentrations of cecal propionate can be increased in young birds. The increase has been used as a metabolic indicator of successful colonization by these probiotic consortia along with a concomitant decrease in the inoculated marker strain of *Salmonella* Typhimurium (Nisbet et al., 1996 a,b). In general, this illustrates some variability in SCFA profiles and concentrations of individual SCFAs, and the potential to modulate their production in the broiler GIT.

Anaerobic fermentations must maintain oxidation-reduction balance with some electron sink source for reducing equivalents generated during energy metabolism (Buckel, 2021). This can be done either by producing reduced end products such as lactate, or reduction of external electron acceptors such as sulfate or nitrate (Buckel, 2021). Some GIT organisms possess hydrogenases that produce hydrogen as a fermentation end product which can be used by methanogens, to produce methane. These organisms are considered primary hydrogen sinks in most animal GITs (Saengkerdsub and Ricke, 2014). Sergeant et al. (2014) identified several uptake hydrogenase sequences in their cecal metagenomic analysis. Still, they were unable to detect the presence of any potential well-known reducing bacteria including sulfate reducers, acetogens, or methanogens. They speculated that organisms such as *Campylobacter* and *Wolinella*, which possess uptake hydrogenases, might serve as the primary hydrogen sinks in the chicken ceca. More recently, Segura-Wang et al. (2021) identified acetogenesis genes in *Lachnospiraceae* strains and *Peptostreptococcaceae* family genomes from broilers based on metagenomic assembled genome analysis. How quantitatively important autotrophic acetogenesis is to hydrogen consumption remains to be determined since at least some acetogens can also use substrates such as glucose for acetate formation (Le Van et al., 1998). The hydrogen consumption role of acetogens in other animal species, such as ruminants, appears to be somewhat dependent on the presence or absence of methanogens. Methanogens can outcompete acetogens for hydrogen when they co-inhabit the GIT and potentially force acetogens to use other substrates (Le Van et al., 1998; Fonty et al., 2007; Li et al., 2020). Whether similar interactions occur between acetogens and methanogens or other hydrogen consuming microbial populations in the poultry GIT remains unknown.

The role of methanogens and hydrogen consumption in the broiler GIT remains unclear as well. Previous culture work indicated that methane is produced from cecal contents, and methanogens have been isolated from several avian fecal contents such as geese, turkeys, and chickens (Miller and Wolin, 1986; Miller and Lin, 2002; Saengkerdsub et al., 2006; Saengkerdsub and Ricke, 2014). Based on molecular analyses, it has been estimated that methanogens may be as much as 3.3% of the total cecal microbiota (Zhu and Joerger, 2003; Qu et al., 2008). Saengkerdsub et al. (2007a.,b) identified the chicken cecal methanogen phylotype, which aligned with *Methanobrevibacter woesei* in the ceca of the young chicks and adult hens. The organism numbers ranged anywhere from approximately 4 to 7 log_10_/gram cecal wet weight with higher numbers in the adult layer hens. How important methanogens are to the overall cecal microbial fermentation remains largely unresolved. Inhibition of methanogens under certain GIT conditions is undoubtedly possible. For example, Sergeant et al. (2014) conducted their metagenomic studies on birds receiving ionophores. Ionophores are known to inhibit methanogens in ruminants, at least in the short term. The decrease in methane activity is usually the result of inhibiting microorganisms producing substrates for methanogens such as hydrogen and formate (McAllister et al., 1996).

## OTHER MICROBIAL METABOLITES PRODUCED BY BROILER GIT MICROBIOTA

**Figure 2:**
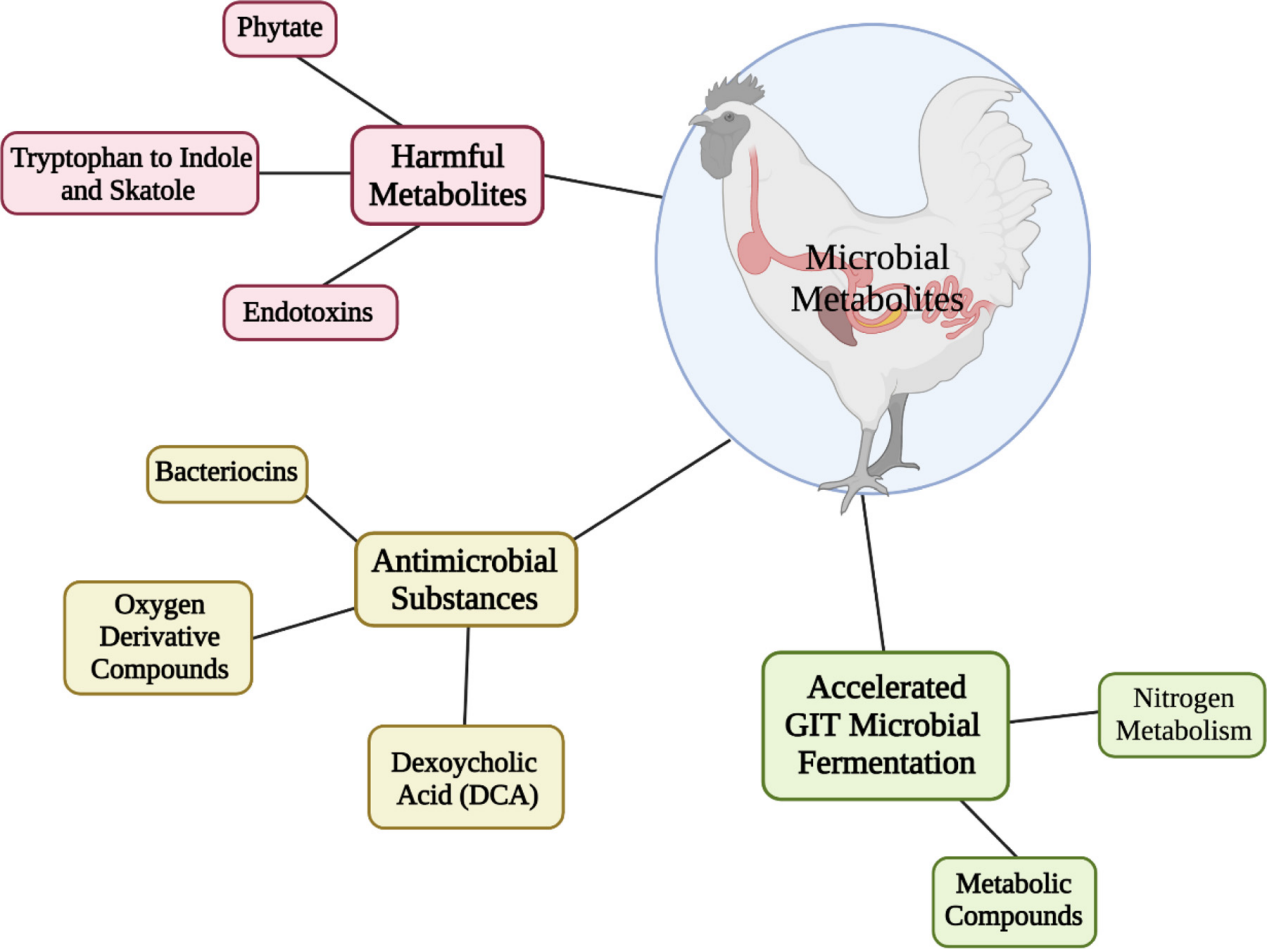
Microbial metabolites produced from microbial fermentation in the gastrointestinal tracts of commercial broilers. Figure created with Biorender.com.

Not all metabolites produced in the poultry GIT are end products directly resulting from fermentation (Figure 2). For example, in addition to the production of SCFA, some bacteria, such as lactobacilli, produce antimicrobial substances referred to as bacteriocins. Bacteriocins are peptide type structures with a variable range of antimicrobial activity against other bacteria with some being broader spectrum than others (Joerger, 2003; Gabriel et al. 2006). While bacteriocin producing lactic acid bacteria are commonly isolated from a wide range of foods, there is much less documentation for actual bacteriocin production occurring in the poultry GIT (Joerger, 2003). There is precedent for bacteriocin production by other animal GIT microorganisms such as rumen bacteria where several different isolates produce bacteriocins and on occasion their presence has been shown to parallel changes in rumen microbial ecology (Russell and Mantovani, 2002). Certain chicken GIT lactobacilli isolates have also been shown to produce bacteriocins. For example, salivaricin SMXD51, a bacteriocin-like compound produced by the cecal isolate *L. salivarius* SMXD51, was effective against *Campylobacter jejuni* and *coli, Listeria monocytogenes, Staphylococcus aureus, Bacillus cereus*, and *Salmonella enterica* and had been shown to reduce *C. jejuni* in broilers when introduced as a probiotic (Messaoudi et al., 2011, 2012; Saint-Cyr. et al., 2017). More recently, Sabo et al. (2020) isolated strains of *Enterococcus faecium* and *Lactococcus lactis* subsp. *Lactis* from broiler ceca that produced bacteriocins effective against *Staphylococcus aureus* and *Salmonell*a Heidelberg. As more of these types of studies are done and the poultry GIT microbiome is characterized in the presence of bacteriocin producing probiotics, it may become more clear whether these compounds can impact the GIT microbial ecology beyond just pathogens. This may also reveal whether these interactions are already occurring in the poultry GIT.

The GIT microbiota can also produce other antimicrobial products such as oxygen derivative compounds. For example, some *Lactobacillus* and *Pediococcus* strains can generate hydrogen peroxide that can be inhibitory to other bacteria (Jin et al., 1997). How important this mechanism is in the poultry GIT remains to be determined, but there is some evidence in other animals that microbial hydrogen peroxide production in the GIT may be active. For example, a hydrogen peroxide overproducing *L. johnsonii* isolate accelerated epithelial cell recovery in mice when the isolate was orally introduced to mice before and during dextran sodium salt induced colitis (Singh et al., 2018).

Other sources of microbial metabolites may serve similar functions in the GIT. Sun et al. (2018) demonstrated that secondary bile acid sodium deoxycholate (**DCA**) generated by orally transplanted microbiota could reduce *C. jejuni* colitis in germ free mice. Further research has demonstrated the effectiveness of this microbially produced metabolite in poultry. Alrubaye et al. (2019) used microbially produced DCA to reduce *C. jejuni* colonization in 28-day-old broilers and modulate the GIT ecology against *C. jejuni* colonization. The application of DCA appeared to possess to selectively alter the poultry GIT microbial population. As such, Alrubaye et al. (2019) demonstrated that DCA supplementation increased the population of Bacteroidetes and decreased Firmicutes while decreasing *C. jejuni* colonization. These results are promising and may have utility for other pathogens, such as *Clostridium perfringens*, in poultry as well. For example, Bansel et al. (2020) used supplementation of deoxycholic acid in broilers to reduce both the inflammation and response to a *C. perfringens*-induced necrotic enteritis (**NE**) infection.

Metabolomic analyses of the chicken GIT have revealed a wide array of metabolic compounds detected in the poultry GIT contents that may have nutritional significance to the host. Rubinelli et al. (2017) characterized the metabolome of *in vitro* cecal incubations in the presence of rice bran. Based on gas chromatograph-mass spectroscopy analyses, 578 total metabolites were detected, of which 211 were identified while 367 remained unknown. Of the compounds associated with rice bran, the authors noted at least a 10-fold increase in malonic acid, ornithine, pantothenic acid, glutamate, and methionine vs. a 20-fold decrease in maltose, among others. Several of these compounds are associated with nitrogen metabolism in the ceca or serve as cofactors for specific metabolic pathways. More recent, *in vivo* broiler studies by Wu et al. (2021) delineated the impact of supplementing a combination of *Pediococcus acidilactici* BBC-1 and xylan oligosaccharides (**XOS**) in commercial broiler diets on the gut metabolome and microbiota. Both the xylan and *Pediococcus* supplements resulted in greater levels of O-acetylserine and the B vitamin pyridoxine while decreasing sorbitol. Collectively, the metabolite data, along with the enrichment of specific metabolism pathways, led the authors to suggest that supplementation of both *Pediococcus acidilactici* BBC-1 and XOS accelerated cecal microbial fermentation (Wu et al., 2021). Accelerated GIT microbial fermentation did align with the increase in butyric acid producing bacteria among the GIT microbiota of the birds fed *Pediococcus acidilactici* BBC-1 and XOS (Wu et al., 2021). It would be interesting to follow up both the metabolomic *in vitro* and *in vivo* studies with a metagenomics assessment to identify prevalent pathways in the poultry GIT that could be attributable to activities directly associated with these particular feed additives.

In addition to generation of beneficial metabolites, poultry GIT bacterial activities can also result in metabolism that is less beneficial to the host and even produce metabolites that are harmful to the host. One aspect often not considered is the fact that some GIT bacteria may in fact compete with the bird for available dietary nutrients in the GIT. Apajalahti and Vienola (2016) have suggested that because the small intestine is dominated by lactobacilli there is active competition between the host and these resident organisms for amino acids, vitamins, and simple sugars. This is because lactobacilli lack the corresponding biosynthetic pathways and thus require a wide range of pre-formed amino acids and B vitamins in synthetic culture media (Morishita et al., 1981). For protein nutrition, Apajalahti and Vienola (2016) have estimated this may be as much as 3 to 6% of the total dietary proteins. This would suggest that lactobacilli probiotics could counteract broiler nutritional efficiency to some extent particularly in the presence of additional amino acid supplementation. However, this may be highly variable since different lactobacilli colonize the small intestine in different regions (Adhikari and Kwon, 2017). Presumably different lactobacilli would possess different nutrient requirements and therefore vary the impact of their presence on the bird. In addition, it is not known how much resident intestinal non-lactobacilli contribute to competitive protein catabolism in the small intestine.

Microbial metabolism of certain dietary constituents can lead to deleterious effects on the bird. This has been observed for both carbohydrate and protein dietary constituents. For carbohydrates, the antinutritive activity of non‐starch polysaccharides in chickens has been attributed by Choct (2002) to increased viscosity, changes in GIT physiology, and the GIT ecosystem. Choct (2002) noted that increased NSPs are accompanied by increased fermentative activity in the small intestine implying that the resident microbial population has been impacted. It has been speculated that this in part may be due to NSP mediated increased viscosity which slows passage rate of digesta to favor a more fermentative microbial population in the small intestine and any effort to decrease viscosity would reverse this relationship (Choct et al., 1996; Choct, 2002). Extensive poultry work with NSP containing diets and inclusion of feed enzymes would tend to support this relationship. For example, Wang et al. (2021) demonstrated that xylanase supplementation of wheat diets fed to broilers decreased ileal diversity, acetate concentrations, and SCFA generating microbiota, but increased lactobacilli. This may also impact other dietary supplementary choices such as selecting prebiotic sources where some are comprised of complex beta-linked polysaccharides which may require more extensive fermentation. Consequently, to avoid microbial imbalances in the small intestine suggests that the best strategy may be to combine these types of prebiotic sources with polysaccharide feed enzymes to assure more substrate availability to the indigenous lactic acid bacterial population. However, more research needs to be done on delineating the impact of passage rate on intestinal microbial composition.

Protein entering the ceca primarily consists of undigested feed protein, endogenous protein such as mucin, epithelial cells, enzymes, and antibodies as well as proteins of microbial origin (Yadav and Jha, 2019). Protein exiting the intestinal tract and entering the ceca can be degraded by cecal bacteria into several potentially toxic metabolites including ammonia, amines, phenols, cresol, and indoles that are deleterious to the host (Apajalahti and Vienola, 2016; Yadav and Jha, 2019). Tryptophan, an essential amino acid, can be metabolized by *Lactobacillus* species and several anaerobic rumen bacteria into indole and skatole, compounds that can negatively impact animal performance and health (Jensen et al., 1995; Bailey et al., 2003; Attwood et al., 2006; Apajalahti and Vienola, 2016). Extent of amine formation may depend on compositional shifts in the cecal microbial population. For example, based on *in vitro* horse cecal incubations, Bailey et al., (2003) concluded that excess carbohydrate in the cecum can lead to the overgrowth of amine producing cecal streptococci and lactobacilli leading to the production of vasoactive amines from amino acids such as tryptophan. Similar shifts could occur in the broiler ceca and increases in specific microorganisms such as lactobacilli may serve as an indicator for predisposition of amine production. This may be of particular concern for diets where fermentable carbohydrates such as certain prebiotics reach the ceca and selectively enrich amine producing lactobacilli. Foodborne pathogens such as *Salmonella* that colonize the ceca also possess an array of decarboxylases for amino acids such as lysine, arginine, and ornithine (Shelaf et al., 1998). It is unclear how much pathogens such as *Salmonella* contribute to cecal amine levels, but it would be of interest to examine amine levels in heavily infected birds. In addition, Gram-negative bacteria can release endotoxins from their lipopolysaccharide (**LPS**) layer during lysis of their cell walls (Gabriel et al., 2006; Ghareeb et al., 2016). These endotoxins can lead to inflammation in broilers and this response has been shown to be reduced by administration of the probiotic Lactobacillus reuteri extracellular vesicles (Hu et al., 2021).

In addition, there are potential harmful compounds that are released from dietary components during the hydrolysis and fermentation activities which are minimally altered or metabolized by the GIT microbiota. For example, based on metabolomic profiles of *in vitro* cecal cultures, Rubinelli et al. (2017) detected increases in several compounds such as 1,2-anhydo-myo-inositol and inositol-4-monophosphate, which they concluded were likely derived from the rice bran phytate. While metabolomic profiles have enhanced the ability to characterize the dynamics of GIT microbial activity, more efforts need to be made to connect microbial composition, functionality, and metabolism. Steps along these lines would help to differentiate compounds that were metabolized or transformed by GIT bacteria in some fashion versus those that were primarily left intact in the presence of GIT microorganisms. Ultimately, identifying microbial pathways and accounting for all substrates and end products via some form of mass balance calculations would provide the means for more precise modeling of the overall microbial ecosystem response to changes in diets and the inclusion of feed additives. However, microbiome compositional analysis is also important as a means to link metabolites with identified taxonomic GIT microbial groups and their predicted metabolic and fermentation pathways. Presumably broiler GIT microbiota compositional differences will likely be reflected in the corresponding metabolism and fermentation pathways predicted for these microorganisms. However, it is less clear how these differences influence the broiler host. It would be assumed that GIT compositional and metabolic profiles would impact the broiler both directly and indirectly. For broiler production, performance metrics such as rate of gain and feed conversion are the commercially relevant responses that must be used to assess the actual GIT microbiome impact on the host.

## BROILER MICROBIOME APPLICATIONS: GROWTH PERFORMANCE

Optimizing broiler performance involves balancing several strategies. These strategies include selective breeding for fast-growing birds and optimized feed conversion to decrease feed costs. Given the importance of GIT physiology for digestion and absorption of dietary components to meet the growth and maintenance requirements of the broilers, the role that the GIT microbiota play in the digestive process is a factor that has a potential impact. With broilers reaching the market at an average of 47 d of age (Dittoe et al, 2020), their microbiota is comparatively less established than laying hens which may be used for egg production for up to 60 wk of age or more (Videnska et al., 2014). Although broilers are raised for less time than layers, age still plays an integral part in establishing the broiler GIT microbiota. This was observed when Lu et al. (2003) followed the development of the ileal and cecal microbiota of commercially reared broilers over time by targeting the 16S rRNA gene. Specifically, Lu et al. (2003) demonstrated that the ileum was different at 3 and 49 d of age but well established between 7 to 21 and 21 to 28 d. In addition, the cecal microbiota was significantly different at 3 and 7 d, 14 to 28 d, and 49 d of age. Differences demonstrated in the ileum and ceca microbiota indicated a successional change in the complexity of the microbiota as the birds matured. In addition, when the birds were 3- and 14-day-old, the microbiota of the ileum and ceca were not different; however, as the birds matured, the ceca became unique in its microbial diversity (Lu et al., 2003). This differentiation among GIT compartments has been shown to hold when indigenous broilers are compared with commercial strains of broilers (Al-Marzooqi et al., 2020a). There is also differentiation within subregions of broiler GIT compartments. For example, Al-Marzooqi et al. (2020b) reported that the microbial populations varied across intestinal segments in Omani chickens. Factors such as poultry breed may need to be considered as well. However, when Montoro-Dasi et al. (2020) compared cecal microbial development between fast- and slow-growing management systems using 2 breeds of broilers, it appeared that the cecal microbial microbiome diversity and taxonomic profiles were relatively resilient to differences in breed. Differences may become more distinct if functionality and metabolite production of the respective cecal microbiota populations in these 2 management systems and actual quantities of the genera present in the ceca were examined.

Another consideration may be the broiler house environment. Takeshita et al. (2021) characterized the microbiota from cecal dropping samples collected at 3 commercial farms to assess the impact of diet phase (starter, grower, and finisher), farm (10,000, 9,000, and 17,000 birds, respectively), and ages of birds (1–6 wk). The specific farm on which the cecal droppings were collected from appeared to have minimal impact on GIT microbial variation based on cecal dropping microbiota diversity assessment. When the age of bird was compared, the richness and diversity of the cecal droppings increased as the broilers matured, which also reflected the change in diet phase (Takeshita et al., 2021). However, when the birds reached the later stages of growth, the cecal-dropping microbiota differences became minimal among the groups of birds, leading the authors to conclude that a level of stabilization had occurred. They also detected an association between the occurrence of *Campylobacter* and differences in cecal dropping OTU abundance in 6-wk-old birds when comparing *Campylobacter* positive and negative samples (Takeshita et al., 2021). They noted that the abundance of *Campylobacter* increased with the bird's age and the change in diet phase.

Age-related differences in poultry GIT microbial diversity have been observed in numerous bird trials and is a factor that has been considered for bird inocula sources to be used for cecal *in vitro* studies (Stanley et al., 2014; Awad et al., 2016; Ballou et al., 2016; Kim et al., 2018; Rychlik, 2020; Feye et al. 2020a). Likewise, age and GIT microbiota are known to impact the appearance of pathogens in the GIT, especially *Campylobacter* (Indikova et al., 2015; Awad et al., 2016; Feye et al., 2020c). As Takeshita et al. (2021) noted, separating the impact of changing diets and the bird's age makes it challenging to delineate their respective individual effects on the changes occurring in GIT microbial composition over the grow-out period of a broiler. In future studies, separating feed versus age differences needs to be examined more in-depth. The strategies may involve designing studies that maintain some broilers on the same feed throughout their life cycle vs. others receiving the more typical changes in feeds during this same period. While such a study could be complex to design, an effort to execute these types of studies may offer an opportunity to differentiate the differences between feed type and age of the bird.

## BROILER MICROBIOME APPLICATIONS: FEED CONVERSION

**Figure 3:**
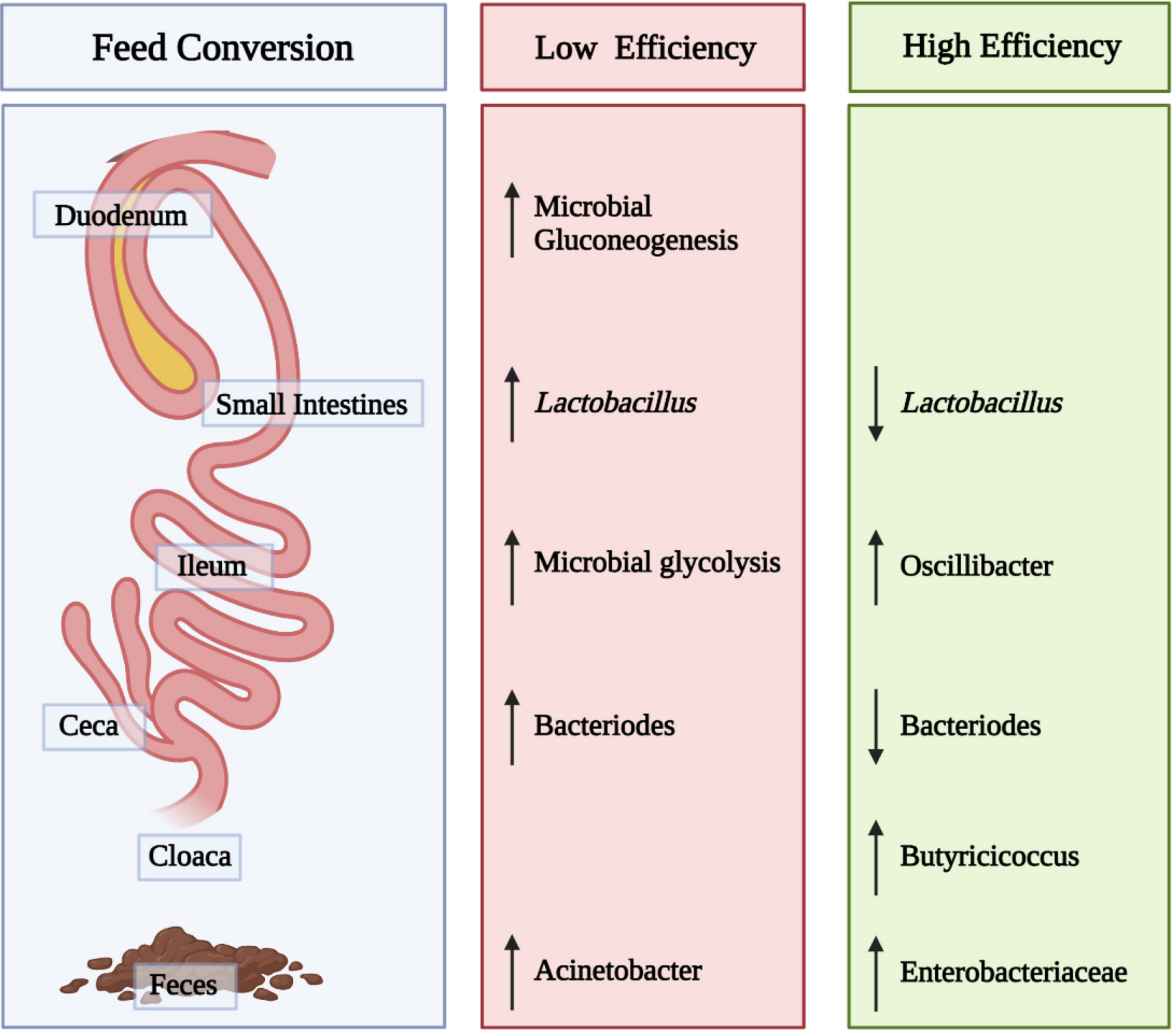
Microbiota and metabolome indicators within the duodenum, ileum, ceca, cloaca, and feces of commercial broilers of low or high feed efficiency (Siegerstetter et al., 2018; Lv et al., 2021, Huang et al., 2021; Liu et al., 2021). Figure created with Biorender.com.

While growth performance is an essential trait for broiler production, feed efficiency is a critical economic factor, particularly when feed costs rise. Consequently, the ability of the broiler bird to maximize conversion of feed into growth and ultimately meat yield is an ongoing research focus. In recent years there have been multiple attempts to link the GIT microbiota with feed digestion and efficiency of nutrient utilization (Figure 3). These studies especially pertain to the ceca, where most of the microbial fermentation occurs, and the upper parts of the broiler GIT. Observations have been made through research that there are variances in the microbiota of high-yielding and low-yielding animals (Huang et al., 2021). Microbiome analysis has been used to delineate bacterial species and metabolic pathways associated with the feed. Because the cecal microbiota may impact some aspects of host nutrient absorption and, in turn, feed efficiency (**FE**), Huang et al., 2021 studied compositional and functional modifications of cecal microbiota between high (**HFE**) and low feed efficiency (**LFE**) groups in yellow broilers. They observed similar microbiota compositions between the HFE and LFE groups; however, the abundances of these microbiota varied between the 2 groups. In addition, within the three main phyla of Firmicutes, Bacteroidetes, and Actinobacteria, genus *Bacteroides* exhibited a significantly higher abundance in HFE than the LFE group and had a negative feed conversion ratio correlation (Huang et al., 2021). Lastly, the study's findings suggest *Bacteroides* may be utilized as a biomarker for FE to enhance growth performance in birds (Huang et al., 2021).

The intestinal microbial population may impact feed efficiency, primarily if nutrients such as dietary amino acids can be utilized by intestinal bacteria and the host (Apajalahti and Vienola, 2016). Lv et al. (2021) sampled the ileum and duodenum of yellow male broilers fed in three stages (d 1 to 20; 21 to 40, and 41 to 63) and euthanized on d 64 for microbiome sequencing. Birds were grouped as either high or low feed efficiency based on feed conversion rate calculations. Taxonomic identification from the microbiome sequencing revealed the dominance of phyla of Firmicutes and Cyanobacteria, and *Lactobacillus, Faecalibacterium*, and *Ruminococcus* genera in the duodenum. In the ileum, the primary phyla were Firmicutes and Proteobacteria long with the genera *Lactobacillus*, SMBB53, and *Enterococcus*. When assessing diversity, the authors concluded that the ileal and duodenal microbial populations of high and low feed efficiency broilers were similar, with the duodenum harboring a more diverse population than the ileum based on alpha diversity estimates. Still, the ileal microbial communities aligned more closely with feed efficiency than their duodenal counterparts. The authors suggested that the ileal microbial glycolysis and the duodenal microbial gluconeogenesis pathways were linked to decreased feed efficiency based on differential functional analysis. Based on taxonomic studies, members of the *Lactobacillus* genus were more significant in the ileal and duodenal populations of low feed efficiency birds than high-efficiency birds leading Lv et al. (2021) to hypothesize that this taxonomic shift may account for the increased glycolysis. Based on the fermentation flexibility of *Lactobacillus* spp. and the diverse subpopulations associated with poultry GIT tract (Adhikari and Kwon, 2017), different *Lactobacillus* species could be predominant depending on location in the small intestine and available substrates from the digesta. This flexibility may partially explain the collective predominance of the genera. In-depth identification of individual species and their fermentation profiles might help correlate taxonomic profiles more precisely with feed efficiency.

Siegerstetter et al. (2018) compared broilers either fed *ad libitum* or restricted-fed with fecal samples collected on d 16 and 29 post-hatch and feed intake measured weekly to estimate residual feed intake (**RFI**). The selected fecal samples were collected within 10 min of being deposited and snap-frozen in liquid nitrogen until DNA extraction and microbiome sequencing were conducted. Groups of birds were classified as either low and high *ad libitum* RFI or low and high RFI restricted fed. When the authors compared fecal microbial populations, they concluded that restrictive feeding exhibited a more significant impact at 29 d post-hatch vs. 16 d post-hatch. It was also noted that there was a trending increase in the microbial richness and evenness of restrictively fed birds vs. those fed *ad libitum*. In addition, network modeling of the microbiome taxa data revealed that *Enterobacteriaceae* were linked with low RFI at 16 d post-hatch. In contrast, *Acinetobacter* was associated with high RFI at both 16- and 29-d post-hatch. When nutrient retention was included in the data analysis, the authors hypothesized that feed intake, substrate availability and host nutritional physiology might be critical factors determining microbial population composition. Their hypothesis was partly based on the observation that the distal intestine and cloacal contents of the *ad libitum* high RFI broilers contained the greatest nutrient quantities. It was further suggested that increased feed retention might influence the relative availability of particular substrates such as nondigested dietary components and, in turn, increase the opportunity for more members of the GIT microbial community to participate in GIT digestion and metabolism. However, if nutrient retention is an essential factor, digesta passage rates would need to be measured in broilers fed feed-restricted or *ad libitum* diets, and these rates compared to GIT microbial growth kinetics and substrate preferences. There are parallels to this hypothesis for interaction between GIT microbial kinetics and retention vs. passage rate in other GIT ecosystems. For example, Russell (1984) has suggested that the retention of a diverse but highly competitive rumen microbial population can be attributed to differences in individual microbial growth kinetics, substrate preferences, and substrate affinities (Russell, 1984). For assessing this interaction in the broiler ceca, continuous cultures containing mixtures of poultry cecal microorganisms and adjusted flow rates could be employed to quantitatively assess the impact on nutrient levels and microbial composition. These culture strategies have been utilized for other GIT microbial ecosystems as well as the selection of competitive exclusion poultry cecal cultures for limiting *Salmonella* (Isaacson et al., 1975; Freter et al., 1983; Rumney and Rowland, 1992; Nisbet et al., 1996a,b, 2000).

In a more recent study, Liu et al. (2021) examined the RFI of broilers individually and characterized the microbial populations in their respective GIT subsections of the ileum, cecum, and cloaca. Broiler chicks were fed 3 diet phases of corn-soybean-based diets consisting of a starter, grower, and finisher diet. Day 35 birds were euthanized to sample their GIT subsections for microbiome analysis on an Illumina HiSeq sequencer. Bioinformatic analyses were conducted to associate RFI responses with identified bacterial taxa. The authors concluded that the majority of those most closely related to low or high RFI belonged to the Clostridiales order, which includes an array of obligate anaerobic microorganisms capable of fermenting indigestible polysaccharides. Among those, they identified enriched levels of *Oscillibacter* in the cecum and *Butyricicoccus* in the cloaca in low RFI broilers that were positively correlated with feed efficiency. *Oscillospira* has been reported as one of the top 5 genera recovered from *in vitro* cecal incubations containing feed and cecal contents (Rubinelli et al., 2017). Members of *Butyricicoccus* have been characterized as high butyrate producers in the broiler cecum, and butyrate is believed to be a promoter of optimal GIT health (Eeckhaut et al., 2008; Bedford and Gong, 2018). Liu et al. (2021) concluded that most of the SCFA producing *Clostridia* were more closely aligned with high RFI broilers and thus represented low feed efficiency. They noted that variation occurred within individual taxa groups. For example, some members of *Lachnospiraceae* were negatively associated with RFI, and others exhibited a positive correlation. As they pointed out, this represents the beginning of in-depth characterization of broiler GIT populations and identifying members of the GIT that correspond most closely to feed efficiency.

Broiler genetics may also factor into the interaction between feed efficiency, digestibility, and GIT microbial populations. Previous studies by Mignon-Grasteau et al. (2004, 2015) demonstrated that poultry digestibility of less digestible diets is genetically heritable, and different GIT microorganisms align with these poultry genetic lines. In a follow-up study, Borey et al. (2020) assessed the 16S rDNA microbial populations of these divergent genetic broiler lines previously selected for either high or low digestive efficiency. The authors' goal was to compare the entire GIT microbial populations of both sets of birds and determine if GIT microbial compositional differences could be detected that matched the differences in digestibility. All birds were fed *ad libitum* low digestible diet containing wheat. A subset of birds was euthanized at 27 d, and contents were removed from the distal ileum, ileocecal junction, jejunum, and the combined contents of both ceca. Overall, they concluded that the combination of genetic selection based on digestibility capacity did influence the entire GIT microbial population. Taxonomic identification revealed *Lactobacillus* as the prevalent genus in the ileum and jejunum and *Faecalibacterium* in the ceca. They noted that while overall alpha and beta diversity estimates could not be linked to digestibility traits, differences in specific OTU profiles were detectable, yielding the highest alignment with digestibility occurring in the cecal microbial populations. This impact of digestibility on the ceca was supported by functional analyses of the microbiome data that revealed nearly double the predicted functions (73) of the cecal microbiota vs. the jejunum (38) and nearly 25-fold those predicted in the jejunum (3). When aligned with the more abundant OTUs and their metabolic characteristics, the authors suggested that at least some of these functions could be responsible for activities such as degradation and subsequent fermentation of nonsoluble polysaccharides that would be expected to originate from these wheat-containing diets. Borey et al. (2020) offered several reasons for this, including more cecal fermentation of a less digestible diet, longer GIT retention times, larger gizzards accompanied by more grinding to decrease particle size digesta, heavier ceca, and differences in intestinal pH. This microbial ecology and feed efficiency relationships are intriguing, and it would be of interest to see how much diet influences the development of these GIT microbial populations as the broiler matures.

Studies that include metabolomic and metagenomic approaches would undoubtedly add additional details to this interpretation and offer opportunities to connect GIT functionality to host feed efficiency responses. Quantitative estimates of individual microorganisms and their metabolism in the GIT may explain why certain organisms prevail under different feed intakes and, in turn, impact feed efficiency. In addition to applying an array of -omic tools, future research effort needs to focus on the development of the broiler GIT during the bird's lifetime as feed type and host GIT development evolve while the broiler matures. It is conceivable that the GIT signature microbial populations associated with feed efficiency may potentially change during the broiler's life cycle. However, this will potentially require collecting data from a more frequent schedule that would involve noninvasive fecal sampling. Identifying potential biomarkers reflective of broiler feed efficiency based on noninvasive approaches would provide the poultry industry with diagnostic tools that can be used for sample collection in the field. However, the question remains how representative a fecal sample is compared to the microbial communities in the broiler GIT (Pauwels et al., 2015; Stanley et al., 2015). Further microbiome comparisons between fecal vs. GIT samples are needed throughout the broiler life cycle to delineate what is being represented in fecal samples and establish some form of standardization for field sampling in commercial broiler houses.

## CONCLUSIONS AND FUTURE DIRECTIONS

Research efforts to connect GIT microbiome with broiler performance have advanced considerably in the past few years. There are several reasons for this. First of all, with the introduction of effective probiotics and other feed additives, it was realized that the broiler GIT microbial population could not only interfere with pathogen colonization but elicit other effects on the bird. Stimulation of the immune system, promotion of GIT health, and nutritional impacts have all been identified as potential outcomes of the interface between the GIT microbiota and the bird host. As these additional factors have become known, efforts to relate GIT microbial composition to broiler performance metrics have received more attention recently. Based on these studies, it appears that there may be some linkages between members of the GIT microbial community and broiler growth rates as well as feed efficiency. However, taxonomic composition characterization alone has not been conclusive. Functional and metabolic activities of the resident GIT microbial population are also important.

In-depth characterization of the broiler GIT microbial population composition and the metabolic activities is an essential next step. For example, certain feed additives can reduce animal mortality, improve GIT and bird health, and restrict the establishment of pathogens, but mechanisms remain to be elucidated. Assessing the constant baseline patterns of broiler production vs. GIT microbial ecology responses can further establish a standard for evaluating newly formulated feed additives. In addition, environmental conditions, feed management, and GIT microbiota metabolic responses are important factors to consider. Continued progress in sequencing resolution along with in-depth data analyses and advanced statistical power to attain associations and network construction of the bacterial populations will help understand host genome wide-microbiome relationships necessary for formulations of feed amendments (Awany et al., 2018). This may require new avenues of research and analytics. For example, the production of germ-free broilers to separate host factors with a controlled introduction of specific GIT microbiota offers a means to distinguish specific GIT microbial factors (Guitton et al., 2020). Advanced analytic tools such as machine learning have been proposed to identify complex associations and develop predictive models for improving food safety and production efficiency (Pitesky et al., 2020). Such analytical approaches also offer an opportunity to integrate the rapidly increasing and complex GIT microbiome compositional database with microbial metabolic and fermentation activities into overall statistical modeling for broiler performance. In conclusion, opportunities for practical and routine use of microbiome data in commercial broiler operations will continue to expand as more metabolite information is collected along with bioinformatic identification of potential microbial fermentation pathways. However, to become more useful as a predictive tool for modeling broiler growth and feed efficiency will necessitate a more quantitative approach. This will involve identifying contributions by individual members of the GIT community to the overall GIT microbial population fermentation profiles. Ultimately, to accomplish this will encompass a combination of quantitative microbiome assessment as well as extensive metabolic stochiometric characterization of individual GIT microbial isolates.

## DISCLOSURES

There are no conflict of interests with any of the authors.
